# The Esterase Gs Derived from *Geobacillus* sp. JM6 Exhibits Hydrolytic Activity on the PET Model Substrates

**DOI:** 10.3390/biology14101387

**Published:** 2025-10-11

**Authors:** Shuyan Duan, Zhaoyi Wei, Yushan Wei, Xiaoyue Cai, Yixuan Liu, Ruiran Fan

**Affiliations:** College of Food Science and Pharmaceutical Engineering, Zaozhuang University, Zaozhuang 277160, China

**Keywords:** PET model substrate, esterase, Gs, hydrolytic activity

## Abstract

**Simple Summary:**

Exploring new polyethylene terephthalate (PET)-degrading enzymes is essential for improving the efficiency of PET degradation. Bis(2-hydroxyethyl) terephthalate (BHET) is a key intermediate in the enzymatic depolymerization of PET. In this study, we investigated, for the first time, the BHET degradation activity and thermal stability of the esterase Gs. Our results indicate that Gs exhibits excellent BHET degradation activity; however, it lacks thermal stability during BHET hydrolysis. We performed a comparative structural analysis of the key amino acids involved in the catalysis of BHET and p-nitrophenyl butyrate (pNPB) by Gs using molecular docking. Gs not only demonstrates strong BHET degradation activity but also degrades the PET model substrate bis(benzyloxyethyl) terephthalate (3PET) and PET nanoparticles. Moreover, the combination of Gs and the mono-2-hydroxyethyl terephthalate (MHET) hydrolase, MHETase, can completely hydrolyze BHET. Finally, considering the structural similarity between LCC-ICCG and Gs, this study presents a novel approach to expanding the search for efficient biocatalysts for the degradation of PET plastic.

**Abstract:**

The continuous increase in demand for polyethylene terephthalate (PET) has drawn global attention to the significant environmental pollution caused by the degradation of PET plastics. Exploring new PET-degrading enzymes is essential for enhancing the degradation efficiency of PET, and esterases and lipases with plastic degradation capabilities have become a focal point of research. In this study, we utilized the ultra-efficient mutant FASTase of the PET-degrading enzyme *Is*PETase, derived from *Ideonella sakaiensis*, as a positive control, based on the similarity in enzyme activity and substrate. We investigated the PET model substrate degradation activities of the esterase Gs and lipase GI, both derived from *Bacillus* spp., as well as the lipase CAI derived from *Pseudomonas* spp. The results indicated that Gs exhibited excellent bis(2-hydroxyethyl) terephthalate (BHET) degradation activity; however, Gs demonstrated a lack of thermal stability when hydrolyzing BHET. Molecular docking analyses were conducted to identify the key amino acids involved in the degradation of BHET by Gs from a structural perspective. At the same time, GI and CAI showed no BHET degradation activity. The combination of Gs and the mono-2-hydroxyethyl terephthalate (MHET) hydrolase, MHETase, can completely hydrolyze BHET, and Gs also exhibited degradation activity against the PET model substrate bis(benzyloxyethyl) terephthalate and PET nanoparticles. Given the structural similarity between PET hydrolase LCC-ICCG and Gs, this study provides new enzyme resources for advancing the efficient biological enzymatic degradation of PET plastics.

## 1. Introduction

The plastic product PET has become an indispensable component in various sectors, including packaging, medicine, aerospace, and agriculture, due to its exceptional physical and chemical properties [1]. However, PET is notoriously difficult to degrade due to the chemical inertness of its ester bonds and aromatic rings [2,3]. Microplastics have been detected in human feces, blood, and placentas, indicating that plastic pollution poses a significant threat to human health and necessitates increased attention [4,5,6,7,8,9]. Compared to traditional physical and chemical processes, using enzymes for biodegradation offers a more environmentally friendly and sustainable method, characterized by lower energy consumption, reduced carbon dioxide emissions, and no groundwater pollution [10,11,12]. However, the lack of or low activity of enzymes that can degrade biodegradable PET plastics has led to the continuous accumulation of these plastic components in the environment [4,5,13].

Although several enzymes with PET degradation activity have been discovered (such as lipases [14,15], esterases [16,17,18,19], and cutinases [20,21,22,23]), they are still insufficient compared to the increasing plastic pollution, highlighting the urgent need to explore more PET-degrading enzymes. PET-degrading enzymes can break down PET into BHET, MHET, terephthalic acid (TPA), and ethylene glycol (EG) [24]. Among these, BHET is one of the main intermediates in the enzymatic depolymerization of PET. Its accumulation not only inhibits the degradation efficiency of PET hydrolases but also increases the difficulty and cost of downstream separation [25]. If enzymes capable of degrading BHET are discovered, they could be used as a basis for fermentation in the biological recycling process of PET waste [26,27]. Therefore, exploring new BHET-degrading enzymes is crucial for improving the degradation efficiency of PET.

**Figure 1:**
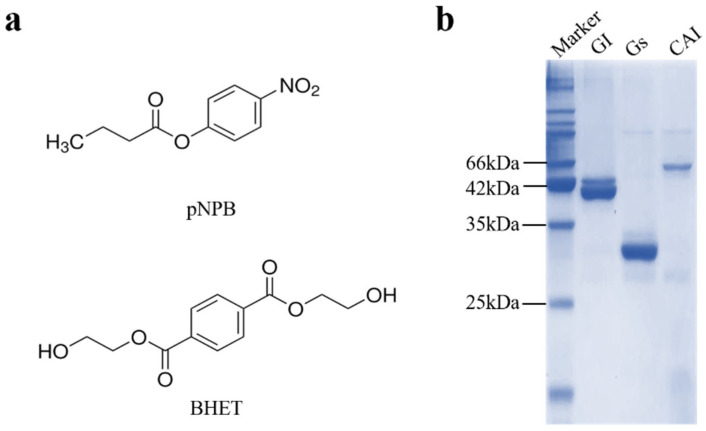
Comparison of enzymatic substrates and protein expression purification. (**a**) Chemical structures of the substrates 4-nitrophenyl butyrate (pNPB) and BHET. (**b**) SDS-PAGE analysis of the protein expression of CAI, GS, and GI.

Both esterases and lipases belong to the α/β hydrolase family and can hydrolyze ester bonds [28]. Different chain lengths of p-nitrophenyl esters serve as model substrates for detecting the activity of esterases and lipases [28,29]. The structure of p-nitrophenyl esters (represented by 4-nitrophenyl butyrate, pNPB) is quite similar to that of BHET (Figure 1), suggesting that there may be enzymes with BHET-degrading activity among esterases and lipases. This study investigated the BHET-degrading activity of esterase Gs and lipase GI from *Bacillus* spp., as well as lipase CAI from *Pseudomonas* spp. The results showed that Gs have strong BHET-degrading activity and can degrade bis(benzyloxyethyl) terephthalate(3PET) and PET nanoparticles, laying the foundation for the subsequent application of Gs in the enzymatic degradation of BHET and further PET degradation.

## 2. Materials and Methods

### 2.1. Materials

The competent cells of *Escherichia coli* BL21(DE3) were obtained from Weidi Biotechnology Co., Ltd. (Shanghai, China). Isopropyl-β-D-thiogalactoside (IPTG) was sourced from Sangon Biotech (Shanghai, China). The polyacrylamide gel rapid preparation kit, universal nuclease, and bacterial active protein extraction reagents were all acquired from Beyotime Biotechnology Co., Ltd. (Shanghai, China). BHET was procured from Sigma (St. Louis, MO, USA), while PET nanoparticles were obtained from ZHONGKEKEYOU (Beijing, China). The 3PET was prepared and supplied by our collaborating laboratory. Proteins Gs (GenBank: AJG36527.1), GI (GenBank: ABC48693.1), and CAI (GenBank: CAI2797360.1) were each linked to the pET21b(+) expression vector and fully synthesized by Sangon Biotech (Shanghai, China). The plasmid pET-21b-FASTase is the laboratory stock.

### 2.2. Bioinformatics Analysis

The prediction of protein molecular weight was conducted using the ExPASy online tool (https://web.expasy.org/compute_pi/). Protein structure prediction was performed using the FastFold online tool (https://www.fastfold.ai/). All protein structure-related alignments and analyses were carried out using PyMOL (https://www.pymol.org). Protein molecular docking was executed using CB-Dock2 online (https://cadd.labshare.cn/cb-dock2/index.php), and ligand compound structures were sourced from PubChem (https://pubchem.ncbi.nlm.nih.gov/). Protein sequence alignment was completed using CLUSTALW online (https://www.genome.jp/tools-bin/clustalw), and the results of the protein multiple sequence alignment were enhanced using ESPript 3.0 online (https://espript.ibcp.fr/ESPript/cgi-bin/ESPript.cgi). Protein gene sequences and amino acid sequence information were retrieved from GenBank (https://www.ncbi.nlm.nih.gov/genbank/).

### 2.3. Protein Expression and Purification

*Escherichia coli* BL21(DE3) cells containing pET-21b-FASTase or Gs, GI, and CAI were cultured overnight in 50 mL of LB medium supplemented with ampicillin (100 μg/mL) at 37 °C and 180 rpm. Subsequently, 20 mL of the overnight culture was transferred to 200 mL of fresh LB medium containing ampicillin and incubated at 37 °C and 180 rpm until the optical density (OD) at 600 nm reached approximately 0.8. The shaker was then cooled to 18 °C, and after about half an hour, when the temperature of the medium stabilized at 18 °C, 0.4 mM IPTG was added to induce the expression of the target protein. The culture was then incubated at 18 °C and 180 rpm for about 20 h.

Subsequently, cells were collected by centrifugation at 3500 rpm for 30 min at 4 °C, and the supernatant was discarded. The cells were lysed using a bacterial protein extraction reagent containing a universal nuclease for 15 min, followed by centrifugation at 8000 rpm for 30 min at 4 °C to remove cell debris. The supernatant was then applied to a nickel affinity chromatography column that had been pre-equilibrated with a buffer containing 50 mM Tris-HCl (pH 8.0), 500 mM NaCl, 10 mM imidazole (pH 8.0), and 5% glycerol. After loading the sample, the protein was eluted sequentially using elution buffers composed of the equilibration buffer, 50 mM Tris-HCl (pH 8.0), 500 mM NaCl, 5% glycerol, with 50 mM imidazole, and 250 mM imidazole (pH 8.0), respectively [30,31]. Finally, the eluted target protein was concentrated to approximately 1 mL using an ultrafiltration tube, and the protein buffer was exchanged for 20 mM Tris-HCl (pH 7.4) and 100 mM NaCl. The concentrated protein was then stored at −80 °C for future use. Throughout the purification process, all samples at each step were analyzed for protein expression levels and purity using 12.5% SDS-PAGE.

### 2.4. Qualitative Detection of Protein BHET Hydrolysis Activity Using a Hydrolysis Ring Experiment

Take 100 mL of a buffer solution containing 20 mM Tris-HCl (pH 7.4) and 100 mM NaCl, and add 2 g of agarose. After autoclaving, allow the temperature to decrease to approximately 60 °C. Then, add 4 mL of a 100 mM solution of BHET dissolved in DMSO, mix thoroughly, and pour the mixture into sterile plates for future use.

Use BHET agar plates to test the hydrolysis activity of the proteins GS, GI, and CAI. Divide one agar plate into five sections, and using a syringe without a needle, create holes in the center of each divided section. Next, add 20 μL of GS, GI, and CAI to three of the sections, while adding 20 μL of the positive control protein FASTase to one section and the negative control buffer (without protein) to the final section. Subsequently, place the plates in a 37 °C incubator, noting the time of placement. Observe and record the changes in the size of the hydrolysis rings every 24 h for a total of 5 days.

### 2.5. Colorimetric Experiment for the Quantitative Detection of Gs BHET Hydrolytic Activity

The method for determining the hydrolytic activity of the enzyme on BHET, based on the phenol red color reaction, has been partially optimized according to the report by Jessica Lusty Beech et al. [32]. For each 200 µL reaction system, the following components were used: 200 nM Gs, 1 mM BHET, 0.1 mM phenol red, 10 mM CaCl_2_, 10% DMSO, and 5 mM HEPES (pH 8.0). A group containing an equal amount of FASTase served as a positive control, while a group with only buffer acted as a negative control. After preparing the reaction system, it was transferred to a 96-well plate and incubated at 37 °C for 18 h to observe and record color changes. Simultaneously, an identical reaction system was prepared, and under the same 37 °C conditions, the absorbance at 550 nm was measured every 15 min using a microplate reader, continuing for the full 18 h. Each experimental group was repeated three times.

### 2.6. Kinetic Measurements

A reaction buffer containing BHET was used as a blank control for instrument calibration and background subtraction. FASTase served as a positive control. The kinetic reaction of Gs hydrolyzing BHET was monitored at 37 °C using the following reaction mixture: Gs at eight concentrations (20 nM, 40 nM, 60 nM, 80 nM, 100 nM, 200 nM, 300 nM, and 400 nM), 1 mM BHET, 0.1 mM phenol red, 10 mM CaCl_2_, 10% DMSO, and 5 mM HEPES buffer (pH 8.0). After 30 min of incubation, the absorbance at 550 nm was measured with a microplate reader.

### 2.7. Colorimetric Experiments to Assess the Synergistic Hydrolytic Activity of Gs and MHETase on BHET

The enzyme reaction system consists of the following components: 0.2 μM Gs, 1 mM BHET, 0.1 mM phenol red, 10 mM CaCl_2_, 10% DMSO, and 5 mM HEPES (pH 8.0). Different concentrations of MHETase are added (0.2 μM, 0.1 μM, 0.05 μM, and 0 μM), and the total volume of the system is adjusted to 200 μL with ultra-pure water. The negative control group does not contain any enzyme, resulting in a total of five groups, each repeated three times. The prepared samples are transferred to a 96-well plate. After all samples are added, a photograph is taken, and the plate is sealed with a sealing film. The absorbance change at a wavelength of 550 nm is measured using a microplate reader, with a detection temperature of 37 °C. The measurements are continuously monitored for 15 h, with readings taken every 15 min. Upon completion of the detection, a photograph is taken to document the changes in OD values and the color changes that occur after the reaction.

### 2.8. Colorimetric Experiment for the Quantitative Detection of the Thermal Stability of Gs Hydrolyzed BHET

Using untreated Gs as a positive control, the thermal stability of the enzyme was evaluated through a colorimetric method after subjecting Gs to temperatures of 80 °C, 90 °C, and 100 °C for 2 h. Each 200 µL reaction mixture consisted of: 200 nM untreated or heat-treated Gs, 1 mM BHET, 0.1 mM phenol red, 10 mM CaCl_2_, 10% DMSO, and 5 mM HEPES (pH 8.0), with a separate group that included only the buffer solution serving as a negative control. Following the preparation of the reaction mixtures, they were transferred to a 96-well plate and incubated at 37 °C for 8 h to monitor color changes. Additionally, an identical reaction mixture was prepared, and the absorbance at 550 nm was measured every 15 min using a microplate reader at 37 °C over the same 8-h period. Each experimental group was conducted in triplicate.

### 2.9. Colorimetric Method for Detecting the Activity of Gs Hydrolyzed PET Nanoparticles

Using FASTase as a positive control and the enzyme-free group as a negative control, the enzyme reaction system is configured as follows: 1 μL of PET nanoparticles (25 mg/mL), 16 μL of Gs, and the reaction mixture is supplemented to a final volume of 200 μL with buffer A (20 mM Tris, 100 mM NaCl), with three replicates for each group. The prepared samples are transferred to a 96-well plate. After all samples have been added, the plate is sealed with a sealing film and incubated at 37 °C for 48 h. Following incubation, the plate is removed, and the absorbance at 600 nm is measured using a microplate reader. The difference in absorbance values between the Gs group and the positive and negative control groups is used to assess whether Gs exhibits PET degradation activity.

### 2.10. Colorimetric Method for Detecting the Hydrolytic Activity of Gs on 3PET

Dissolve 20 mg of 3PET in 0.6 mL of acetone. In a fume hood, add the dissolved 3PET dropwise to 10 mL of preheated 50 mM Tris buffer (pH 8.0), while continuously shaking during the addition. After heating and stirring the mixture in the fume hood for 2 h, centrifuge it to collect the supernatant, which constitutes the prepared 3PET emulsion. Use an equal volume of FASTase as a positive control and a group without added enzyme as a negative control to conduct the Gs hydrolysis activity test on 3PET. The enzyme reaction system is as follows: 190 μL of 3PET emulsion and 10 μL of Gs, with each sample group repeated three times. Transfer the prepared samples to a 96-well plate, seal the plate with a sealing film after all samples are added, and take a photograph for record-keeping. Set the temperature of the microplate reader to 37 °C, and measure the absorbance at a wavelength of 580 nm, continuously monitoring for 4.5 h and measuring every 15 min. After the detection is complete, take another photograph for record-keeping.

## 3. Results and Discussion

### 3.1. Comparison of Enzymatic Substrates and Protein Expression Purification

According to existing reports, the three proteins CAI, GS, and GI all exhibit hydrolytic activity on model substrates composed of nitrophenyl esters [33,34,35]. The structure of nitrophenyl esters, represented by 4-nitrophenyl butyrate (pNPB), closely resembles that of BHET (Figure 1a). Prior research suggests that enzymes active on nitrophenyl esters can also act on BHET or PET model substrates [36]. Therefore, we hypothesize that these three proteins may also exhibit hydrolytic activity toward BHET. To test this hypothesis, bioinformatics analyses and expression purification were initially performed on CAI, GS, and GI. Based on the amino acid sequences of the three proteins, the theoretical molecular weights were calculated to be 50 kDa for CAI, 29 kDa for GS, and 41 kDa for GI. After purification, proteins with approximately 90% purity were obtained at their respective molecular weights (Figure 1b). The proteins were concentrated, exchanged into buffer A, and subsequently stored at −80 °C for further studies on their enzymatic properties.

### 3.2. The Qualitative Detection Results from the Hydrolysis Zone Experiment Indicate That Gs Exhibits BHET Hydrolytic Activity

**Figure 2:**
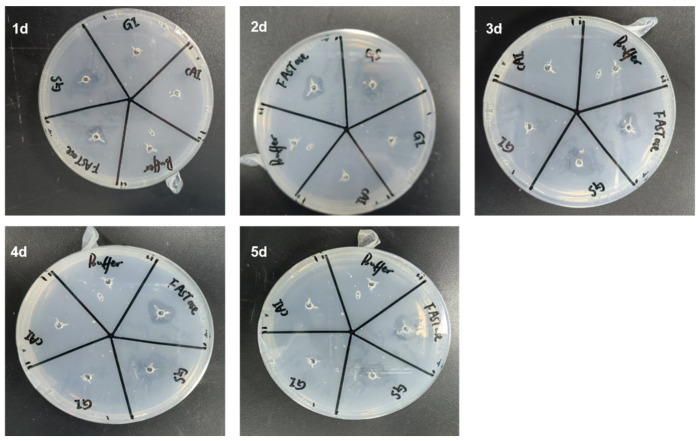
Qualitative detection of BHET hydrolytic activity in proteins using hydrolysis ring assays conducted over five consecutive days. Here, 1d, 2d, 3d, 4d, and 5d represent the first, second, third, fourth, and fifth days, respectively.

Using BHET agar plates, the protein-hydrolytic activity of BHET was continuously assessed over five days. The size of the hydrolysis zone indicated that Gs exhibited BHET hydrolytic activity comparable to the positive control, FASTase [37], while the other two proteins, GI and CAI, demonstrated no BHET hydrolytic activity (Figure 2). Notably, from days one to four, the hydrolysis zone gradually increased in size with the extension of the reaction time; however, by day five, there was no significant difference in the size of the hydrolysis zone compared to day four (Figure 2). It is speculated that, by day five, the enzyme activity had significantly diminished. This observation aligns with previous reports indicating that maintaining enzyme activity necessitates the timely supplementation of fresh plastic-degrading enzymes [38].

### 3.3. The Results of the Colorimetric Experiment Indicate That Gs Exhibits Hydrolytic Activity on BHET

The most established method for detecting PET degradation products is currently high-performance liquid chromatography [38,39,40]. This technique is accurate and sensitive but has limitations regarding speed, throughput, and ease of measuring enzyme kinetics. An absorbance-based method for detecting PET degradation products offers a simple, fast, low-volume, and resource-efficient approach for monitoring enzyme kinetics [41].

**Figure 3:**
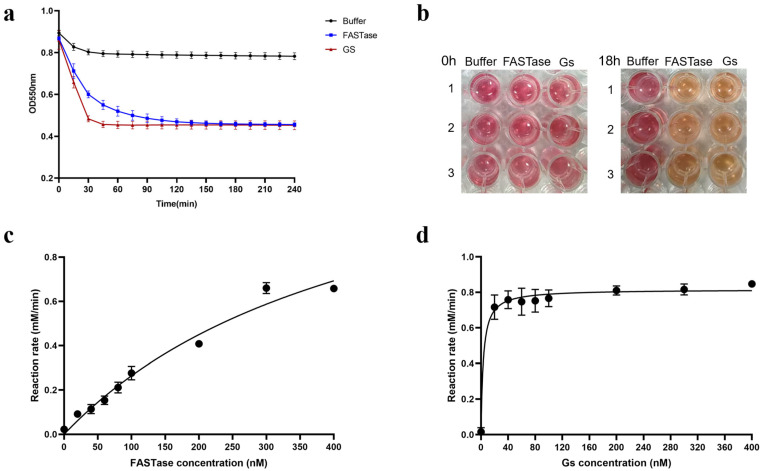
Colorimetric experiments for the quantitative detection of Gs BHET hydrolysis activity. (**a**) Colorimetric assay for the quantitative detection of Gs BHET hydrolysis activity, accompanied by the corresponding color change shown in (**b**). (**c**) Kinetic measurements of FASTase over 30 min. (**d**) Kinetic measurements of Gs over 30 min.

The results of the colorimetric method for quantitative detection indicate that Gs exhibits BHET hydrolysis activity nearly identical to that of the reported ultra-efficient PET hydrolase, FASTase. Furthermore, in comparison to FASTase, Gs demonstrates a higher hydrolysis efficiency, as it is nearly completely hydrolyzed within one hour of the reaction’s initiation. At the same time, FASTase requires about three hours to achieve complete hydrolysis (Figure 3a). The corresponding color change of the phenol red indicator in this method further confirms that Gs possesses strong BHET hydrolysis activity; as Gs hydrolyzes BHET, terephthalic acid is produced, resulting in a color shift of the reaction system from red to orange-yellow. Ultimately, the color of the Gs treatment group closely resembles that of the FASTase treatment group (Figure 3b).

Previous studies have demonstrated that the absorbance method can detect changes in the catalytic rates of plastic-degrading enzymes [41]. In this study, we applied absorbance spectrophotometry to assess the enzyme kinetics of Gs and FASTase. First, a standard curve of absorbance values corresponding to the product was generated (Figure S1). The absorbance values measured from the reaction were then used to calculate the product concentration by referencing this standard curve. Next, the enzyme reaction rate was determined. Using the Michaelis-Menten equation in GraphPad Prism 8, the kinetic curves for Gs and FASTase catalyzing BHET were fitted, as shown in Figure 3c,d. Based on the software-fitted kinetic curves, the maximum reaction velocity (Vmax) of Gs was determined to be 0.8159 mM/min, with a substrate affinity (Km) of 3.382 nM. For FASTase, the maximum reaction velocity (Vmax) was 1.511 mM/min, and the substrate affinity (Km) was 472.4 nM (Table S1). These results indicate that, compared to FASTase, Gs catalyzes the reaction system to reach its maximum reaction rate more rapidly and exhibits a stronger binding affinity for BHET (Figure 3c,d).

### 3.4. The Results of the Colorimetric Experiment Indicate That Gs Does Not Possess the Hydrolytic Thermal Stability of BHET

**Figure 4:**
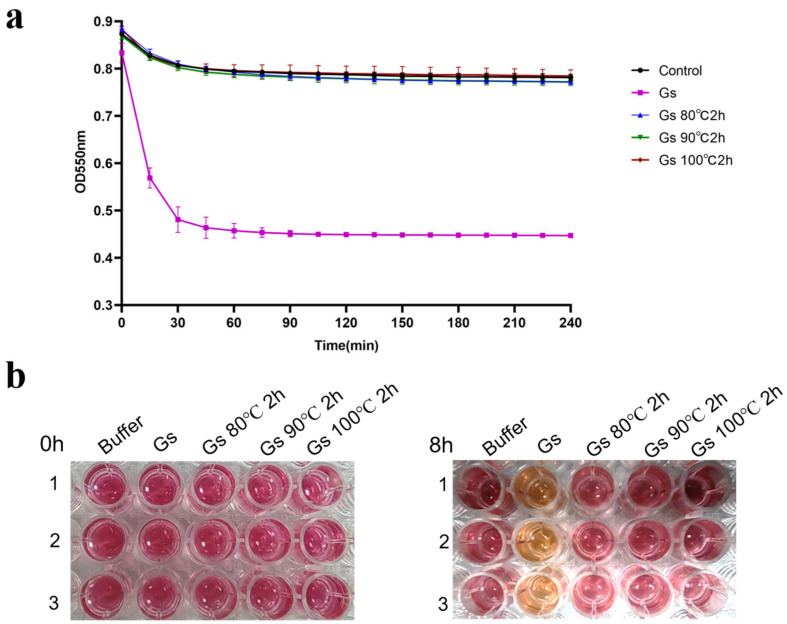
(**a**) Colorimetric experiment for the quantitative detection of Gs BHET hydrolysis thermal stability, accompanied by the corresponding color change (**b**).

The results of the colorimetric quantitative detection indicate that Gs no longer exhibits BHET hydrolysis activity after being treated at 80 °C, 90 °C, and 100 °C for 2 h. This finding suggests that Gs lacks significant thermal stability when hydrolyzing BHET (Figure 4a). Additionally, the absence of color change in the phenol red indicator before and after the reaction further confirms that Gs has lost its BHET hydrolysis activity following heat treatment (Figure 4b). In contrast, previous reports indicated that Gs retained high hydrolysis activity when catalyzing pNPB after being heat-treated at 100 °C for 18 h [34]. This discrepancy suggests that Gs may employ different catalytic mechanisms when hydrolyzing BHET compared to pNPB.

### 3.5. Molecular Docking of the Gs-BHET Complex

**Figure 5:**
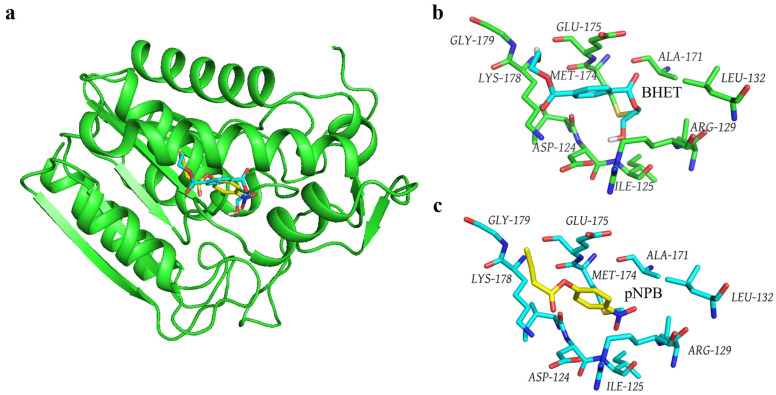
(**a**) Structural model of the Gs-BHET and Gs-pNPB molecular docking complexes. Blue indicates the substrate BHET, while yellow indicates the substrate pNPB. (**b**) Gs amino acids located within 4 Å of BHET. Blue represents the substrate BHET. (**c**) Gs amino acids located within 4 Å of pNPB. Yellow represents the substrate pNPB.

To compare the mechanism of Gs in catalyzing BHET and pNPB, molecular docking was employed to obtain the structures of the Gs-BHET and Gs-pNPB complexes (Figure 5a). Structural comparisons indicate that Gs binds to both BHET (Figure 5b) and pNPB (Figure 5c) within the same binding pocket. An analysis of the amino acids within a 4 Å range of BHET (Figure 5b) and pNPB (Figure 5c) reveals that the amino acids surrounding both substrates are identical. This finding contradicts the hypothesis that Gs loses its BHET hydrolysis activity after heat treatment while still maintaining high hydrolysis activity for pNPB after being subjected to 100 °C for 18 h, suggesting that the difference in catalytic mechanisms may be the underlying cause. In the future, this issue can be further investigated by analyzing the crystal structures of the Gs-BHET and Gs-pNPB complexes.

### 3.6. Comparison of the Structure of Gs and Other BHET Hydrolases

**Figure 6:**
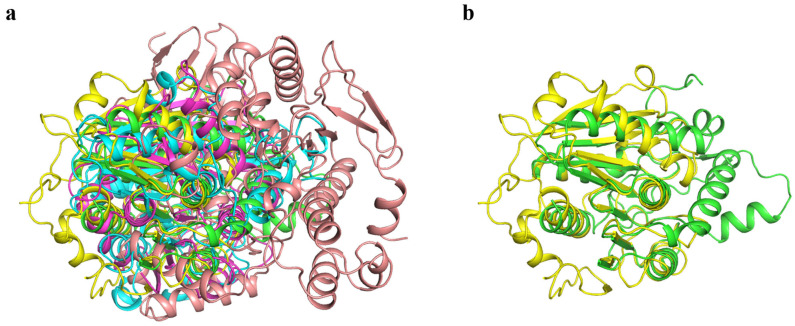
(**a**) Structural alignment of Gs with other BHET hydrolases (green: Gs; yellow: LCC-ICCG; blue: CALB; magenta: FASTase; pink: BsEst). (**b**) Structural alignment of Gs and LCC-ICCG (green: Gs; yellow: LCC-ICCG).

BHET hydrolase is generally not recognized as a distinct enzyme category but rather serves as a collective term for various enzymes exhibiting this function. Currently, the BHET hydrolases that have been discovered or extensively studied primarily originate from microorganisms, such as bacteria and fungi [25]. Based on their sources and characteristics, these enzymes can be classified into esterases, lipases, cutinases, and PET hydrolases that demonstrate BHET hydrolytic activity [42]. To compare the structural similarities and differences between Gs and other BHET hydrolases, we performed structural alignments of Gs with representative members of esterases, lipases, cutinases, and PET hydrolases known to possess BHET hydrolytic activity. Specifically, we selected the esterase BsEst [25], lipase CALB [43], cutinase LCC-ICCG [38,44], and PET hydrolase FASTase [37] for structural comparison with Gs (Figure 6a). The structural alignment results revealed that the RMSD values between BsEst, CALB, LCC-ICCG, FASTase, and Gs were 19.852 Å, 6.942 Å, 1.384 Å, and 18.401 Å, respectively. Among these, LCC-ICCG exhibited the highest structural similarity to Gs, with an RMSD of 1.384 Å (Figure 6b). LCC-ICCG is a reported cutinase with hydrolytic activity toward various PET substrate types, suggesting that Gs likely also possesses hydrolytic activity toward multiple PET substrates. In contrast, Gs and FASTase showed significant structural differences, with an RMSD of 18.401 Å. This finding suggests that Gs, as a novel BHET-degrading enzyme, likely has a specific and unique catalytic mechanism distinct from that of FASTase.

### 3.7. The Synergistic Action of Gs and MHETase Can Completely Hydrolyze BHET

**Figure 7:**
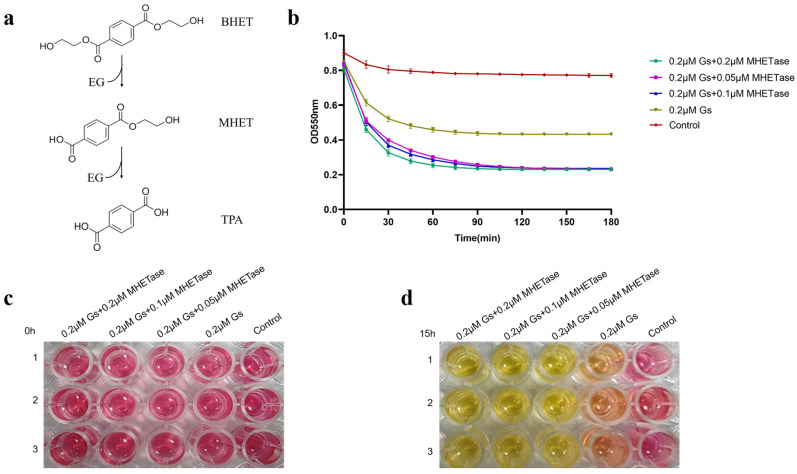
Qualitative and quantitative assessment of the effect of MHETase on the hydrolytic activity of Gs on BHET. (**a**) Schematic diagram illustrating the hydrolysis reaction of BHET. (**b**) Quantitative analysis of the impact of MHETase on the hydrolytic activity of Gs on BHET, utilizing the phenol red colorimetric method. Qualitative evaluation of the effect of MHETase on the hydrolytic activity of Gs on BHET, employing the phenol red color method, with observed color changes after 0 h (**c**) and 15 h (**d**) of reaction.

The colorimetric method was employed to detect the synergistic hydrolysis of BHET activity by Gs and MHETase. As illustrated in Figure 7a, the hydrolysis of BHET produces the intermediate product MHET. If the reaction proceeds to completion, the final products will consist solely of TPA and EG; however, if the reaction is incomplete, the products will include MHET, EG, and TPA. To ascertain whether Gs can catalyze the complete hydrolysis of BHET, we established MHETase, which can hydrolyze MHET, as a control group for the experiment. The phenol red colorimetric method was utilized to determine whether MHETase facilitated Gs in further hydrolyzing BHET, specifically to assess if Gs can completely hydrolyze BHET. In this method, a lower OD550 nm value indicates a more complete hydrolysis of BHET, resulting in increased TPA production.

The accompanying color change of phenol red signifies that, when there is no hydrolysis of BHET, the color is red; when both MHET and TPA are produced, it appears orange-red; and when hydrolysis is complete, yielding only TPA, it turns yellow. The results presented in Figure 7b demonstrate that when 0.2 μM, 0.1 μM, and 0.05 μM of MHETase were added, the trends in OD value changes and the final values were nearly identical. More TPA was produced following the addition of MHETase, resulting in a lower OD550 nm value compared to the group without MHETase. This indicates that Gs cannot completely hydrolyze BHET. The qualitative detection of phenol red further supports this conclusion: at 0 h of reaction, all five groups were red (Figure 7c); after 15 h of reaction, the groups containing both Gs and MHETase turned yellow, while the group with only Gs appeared orange-yellow (Figure 7d). This suggests that the addition of MHETase is essential for the reaction to reach completion, indicating that Gs and MHETase work synergistically to fully hydrolyze BHET.

### 3.8. Gs Exhibits Hydrolytic Activity Towards 3PET and PET Nanoparticles

**Figure 8:**
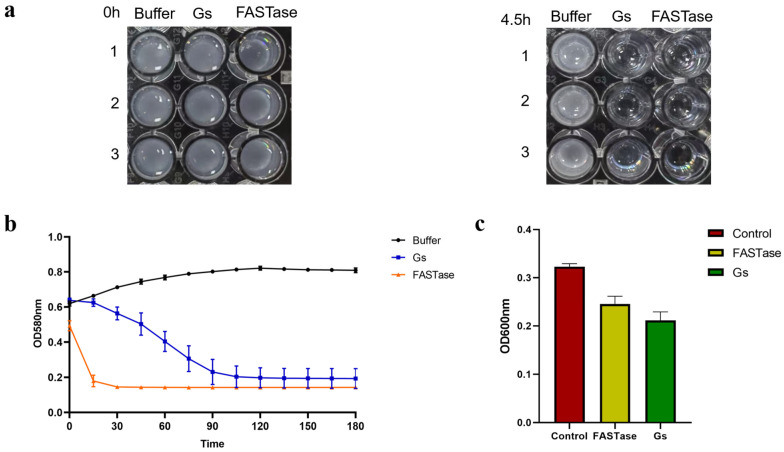
Active detection of Gs-hydrolyzed 3PET and PET nanoparticles. (**a**) Color change before and after Gs hydrolysis of the 3PET emulsion. (**b**) Colorimetric method for the quantitative detection of absorbance changes before and after Gs hydrolysis of the 3PET emulsion. (**c**) Colorimetric method for the quantitative detection of absorbance changes before and after Gs hydrolysis of PET nanoparticles.

The colorimetric method was employed to assess the degradation activity of Gs on 3PET. In this approach, a stronger turbidity of the 3PET emulsion corresponds to a higher absorbance value; conversely, lower turbidity results in a lower absorbance value. If the protein sample is capable of degrading the 3PET emulsion, the corresponding absorbance value will decrease. As illustrated by the turbidity changes in Figure 8a, both Gs and FASTase demonstrate hydrolytic activity on 3PET when compared to the negative control group. Following hydrolysis, the 3PET emulsion in both experimental groups became clear, while the negative control group remained an emulsion. Figure 8b indicates that the experimental group with Gs exhibited a final absorbance value slightly lower than that of the positive control group, FASTase; however, the degradation rate of Gs was significantly slower than that of FASTase.

FASTase was completely degraded within 15 min, whereas Gs required approximately 120 min to achieve a similar effect (Figure 8b). This suggests that Gs possesses 3PET degradation activity, albeit weaker than that of the positive control group. Subsequently, the colorimetric method was further utilized to evaluate whether Gs exhibits hydrolytic activity on PET nanoparticles. In this method, a stronger turbidity of the PET nanoparticles correlates with a higher absorbance value; conversely, lower turbidity results in a lower absorbance value. If the protein sample can degrade PET nanoparticles, the corresponding absorbance value will decrease. As shown in Figure 8c, the experimental group with Gs displayed the lowest absorbance value compared to both the negative and positive control groups, indicating that Gs possesses PET nanoparticle degradation activity, which is slightly superior to that of the positive control group.

## 4. Conclusions

Exploring new enzymes capable of degrading BHET is crucial for improving the efficiency of PET degradation [26]. In this study, we investigated, for the first time, the BHET degradation activity and thermal stability of the esterase Gs. Our results indicate that, compared to FASTase, Gs catalyzes the reaction system to reach its maximum reaction rate more rapidly and exhibits a stronger binding affinity for BHET. However, Gs lacks thermal stability during BHET hydrolysis. We performed a comparative structural analysis of the key amino acids involved in the catalysis of BHET and pNPB by Gs using molecular docking. Gs demonstrates strong BHET degradation activity and also degrades the PET model substrate 3PET as well as PET nanoparticles. Finally, given the structural similarity between LCC-ICCG and Gs, this study offers a novel approach to expanding the search for efficient biocatalysts for PET plastic degradation by exploring new plastic-degrading enzymes based on substrate similarity.

## Data Availability

The original contributions presented in this study are included in the article/Supplementary Materials. Further inquiries can be directed to the corresponding author.

## Supplementary Materials

The following supporting information can be downloaded at: https://www.mdpi.com/article/10.3390/biology14101387/s1. Figure S1: Product Corresponding Absorbance Standard Curve; Table S1: Michaelis-Menten.
