# Supplementary material for: The Esterase Gs Derived from Geobacillus sp. JM6 Exhibits Hydrolytic Activity on the PET Model Substrates
Source: Biology (Basel). 2025 Oct 11;14(10):1387. doi: 10.3390/biology14101387 (PMC12562035; doi:10.3390/biology14101387)
Supplement: Supplementary file 1 [file biology-14-01387-s001.zip › biology-3844706-supplementary.pdf]

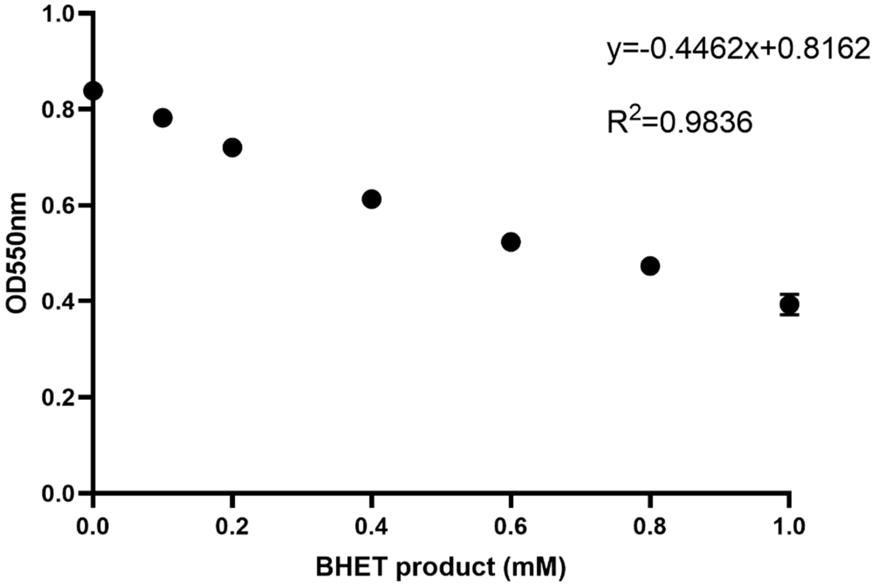

Figure S1. Product Corresponding Absorbance Standard Curve.

Table S1. Michaelis-Menten.

**Michaelis-Menten**	**Gs**	**FASTase**
**Best-fit values**		
Vmax (mM/min)	0.8159	1.511
Km (nM)	3.382	472.4
95% CI (profile likelihood)		
Vmax (mM/min)	0.7848 to 0.8482	1.217 to 2.021
Km (nM)	1.307 to 5.755	332.1 to 725.5
**Goodness of Fit**		
Degrees of Freedom	25	25
R squared	0.9656	0.9733
Sum of Squares	0.05600	0.03642
Sy.x	0.04733	0.03817
**Constraints**		
Km	Km > 0	Km > 0
